# Supplementary material for: Exploitable mechanisms of antibody and CAR mediated macrophage cytotoxicity
Source: Nat Commun. 2025 Jul 1;16:5616. doi: 10.1038/s41467-025-60745-x (PMC12216399; doi:10.1038/s41467-025-60745-x)
Supplement: Supplementary file 1 — Supplementary Information [file 41467_2025_60745_MOESM1_ESM.pdf]

Supplementary information for

Exploitable Mechanisms of Antibody and CAR Mediated Macrophage Cytotoxicity

Authors:

Tianyi Liu[1,2], Meng Zhang[1,2], Tatyanah Farsh[1,2], Haolong Li[1,2], Audrey Kishishita[3], Abhilash Barpanda[4], Stanley G. Leung[1,2], Jun Zhu[1,2], Hyuncheol Jung[5,6], Junjie Tony Hua[1,2], Xiaolin Zhu[1,6], Alexander B. Kim[7,8], Young Ah Goo[9], Minsoo Son[9], Jaenyeon Kim[9], Aish Subramanian[1,2], Martin Sjöström[2,19,20], Katherine C. Fuh[1,10], Jocelyn S. Chapman[1,10], Julia Carnevale[1,5,6,11] Luke A. Gilbert[1,12,13], Aparna Lakkaraju[14,15], Peter M. Bruno[1,12], David Quigley[1,16], Arun P. Wiita[4,17,18], Felix Y. Feng[1,2,12,*] and Carl J. DeSelm[7,8,21,*]

Correspondence: deselmc@wustl.edu (C. J. D.)

Supplementary Figure 1. Generation and characterization of CAR macrophages (related to Figure 1).

Supplementary Figure 2. In vitro validation of hits nominated from macrophage co-culture CRISPR screens (related to Figure 2).

Supplementary Figure 3. Lower ATG9A mRNA is associated with better survival in patients with cancer (related to Figure 2).

Supplementary Figure 4. Lower ATG9A mRNA (≥ median) is associated with better survival in several additional cancer types (related to Figure 2).

Supplementary Figure 5. ATG9A protects ovarian cancer cells from CAR-M-induced plasma membrane damage. (related to Figure 2 and 3).

Supplementary Figure 6. Roles of ATG9A in other pathways involved in cancer cell response to macrophages (related to Figure 5).

Supplementary Figure 7. ATG9A KO increases the number and size of lipid droplets in OVCAR-8 cells (related to Figure 6).

Supplementary Figure 8. Single-cell RNA-Seq reveals upregulated pro-inflammatory and reduced anti-inflammatory macrophage signatures in ATG9A KO tumors (related to Figure 9).

Supplementary Figure 9. Gating strategies used for Flow cytometry or cell sorting. (related to Figure 2 and Figure 9).

Supplementary Table 1. Summary of cell lines and animal models used in this study.

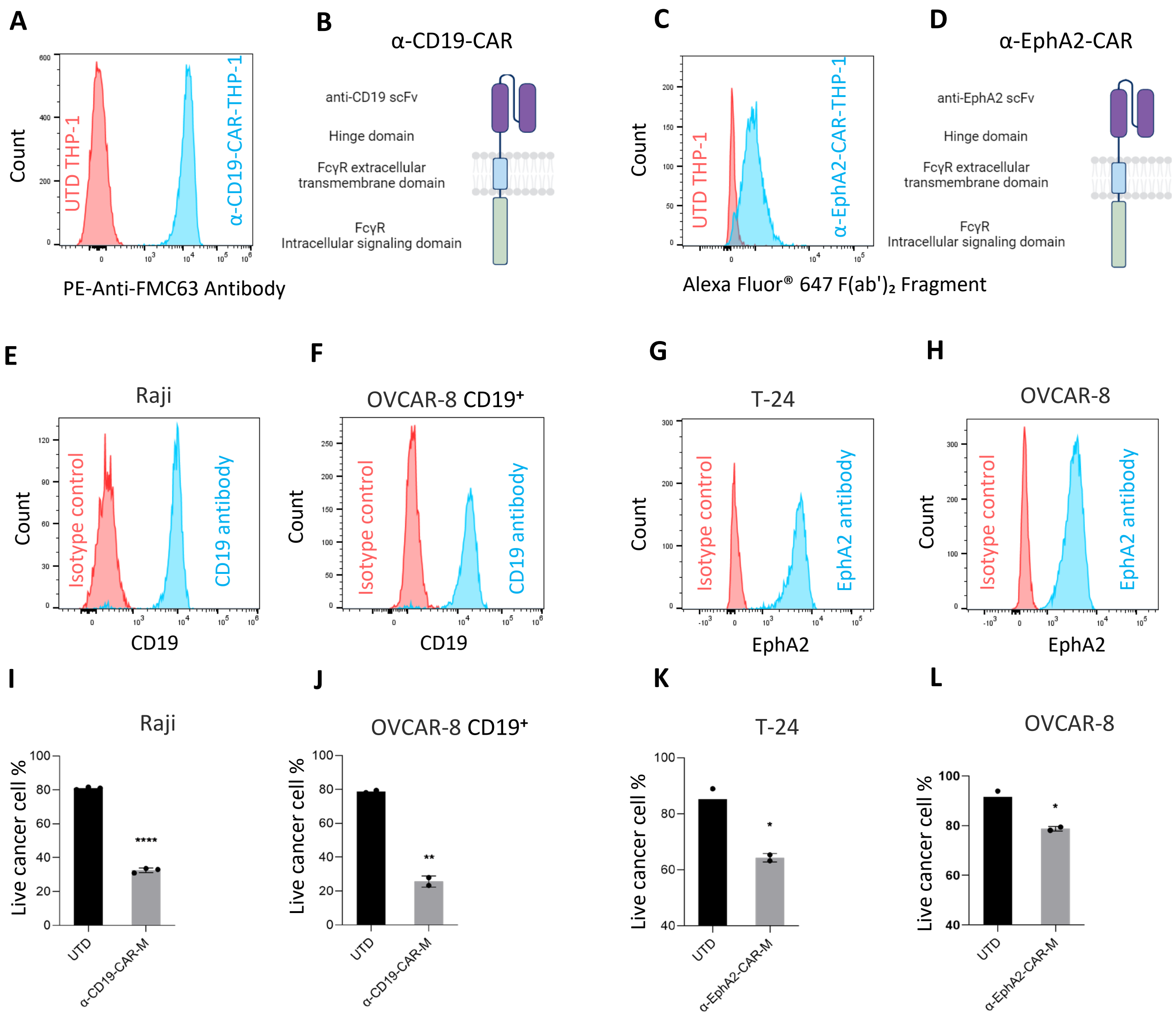

Supplementary Figure 1. Generation and characterization of CAR macrophages (related to Figure 1).
(A) Flow cytometry staining to confirm α-CD19-CAR expression on THP-1 cells. (B) α-CD19-CAR construct. (C) Flow cytometry staining to confirm α-EphA2-CAR expression on THP-1 cells. (D) α-EphA2-CAR construct. (E-F) Confirmation of CD19 expression on Raji cells and $CD19^+$ OVCAR-8 cells by flow cytometry. (G-H) Confirmation of EphA2 expression on T-24 cells and OVCAR-8 cells by flow cytometry. (I-J) Raji cells and $CD19^+$ OVCAR8 cells were co-cultured with α-CD19-CAR-THP-1 macrophages or untransduced (UTD) THP-1 macrophages for three days. Live cancer cell % was calculated by normalizing the day-three cell count in the co-cultured group by the cancer-cell-only group (n = 3 biological replicates; mean ± SEM). (K-L) T-24 cells and OVCAR-8 cells were co-cultured with α-EphA2-CAR-THP-1 macrophages or UTD THP-1 macrophages for three days. (n=3 biological replicates; mean ± SEM). Statistical significance was determined using two-tailed unpaired Student's t-tests. *P < 0.05; **P < 0.01; ***P < 0.001.

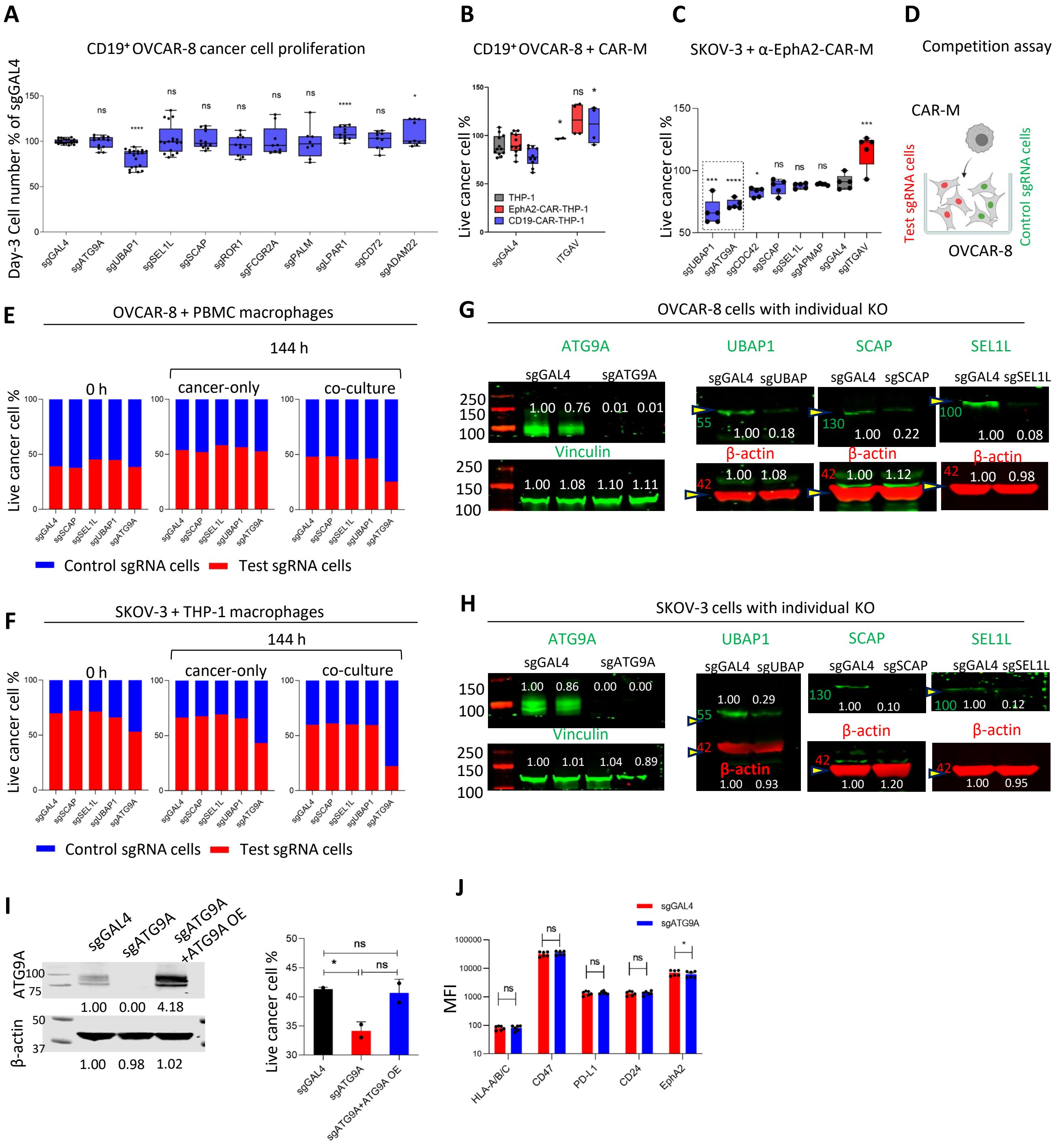

Supplementary Figure 2. In vitro validation of hits nominated from macrophage co-culture CRISPR screens (related to Figure 2). (A) Proliferation of OVCAR-8 cells over three days after individual guides KO (n = 6 biological replicates; mean ± SEM). Statistical significance was determined using two-way ANOVA with Tukey's multiple comparisons test. (B) Control or sgITGAV CD19⁺ OVCAR-8 cells were cultured alone or with parental, α-EphA2-CAR or α-CD19-CAR macrophages for three days. (n = 4 biological replicates; mean ± SEM). Statistical significance was determined using two-way ANOVA with Tukey's multiple comparisons test. (C) Individual guides KO co-culture experiments with SKOV-3 cells. (n = 5 biological replicates; mean ± SEM). Statistical significance was determined using two-way ANOVA with Tukey's multiple comparisons test. (D) Competition assay schematic. (E) Competition assay using OVCAR-8 cells and PBMC macrophages. (F) Competition assay using SKOV-3 cells and THP-1 macrophages. (G-H) Individual KO efficiency confirmation by Western blot. (I) Western blot showing ATG9A expression in OVCAR-8 cells with control sgRNA (sgGAL4), ATG9A knockout (sgATG9A), or sgATG9A cells reintroduced with ATG9A overexpression (sgATG9A + ATG9A OE). Right: percentage of live cancer cells after 3-day co-culture with α-EphA2 CAR macrophages (n = 3 biological replicates; mean ± SEM). Statistical significance was determined using one-way ANOVA with Dunnett's multiple comparisons test. (J) Summary of phagocytosis checkpoint expression on control and sgATG9A OVCAR-8 cells by flow cytometry (n =6 biological replicates; mean ± SEM). Statistical significance was determined using two-tailed unpaired Student's t-tests for each protein expression. *P < 0.05; **P < 0.01; ***P < 0.001. Some elements of this figure were created with BioRender.com and are included under a publication license in accordance with BioRender's user agreement.

A

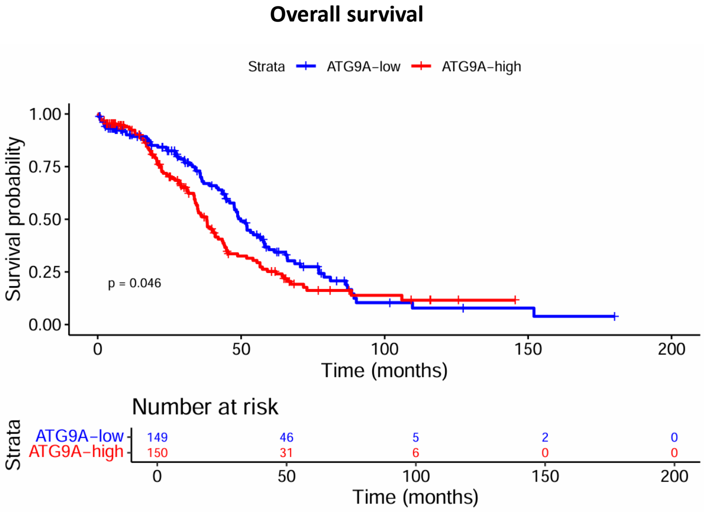

B

Z-scores from TCGA RNA-Seq

Gene	Pan-Cancer	Ovarian cancer
ATG9A	4.68	1.83
ROR1	3.4	0.47
FCGR2A	3.38	1.47
CD72	1.3	-0.35
LPAR1	1.21	1.97
SCAP	-0.23	0.03
PALM	-0.98	0.51
SEL1L	-1.29	0.02
ADAM22	-2.16	0.65
UBAP1	-2.31	-0.54

Z-Score -5 -4 -3 -2 -1 0 1 2 3 4 5

Supplementary Figure 3. Lower ATG9A mRNA is associated with better survival in patients with cancer (related to Figure 2). (A) Kaplan-Meier survival curves showing that lower ATG9A mRNA levels (≥ median) are associated with better overall survival in patients with ovarian serous cystadenocarcinoma. (B) Pan-cancer RNA-Seq Z-scores of hits nominated from the screen. The Z-scores were calculated by combining individual RNA-Seq Z-scores for each cancer type using Stouffer's method. Positive Z-scores indicate an association with decreased survival, while negative Z-scores indicate an association with increased survival.

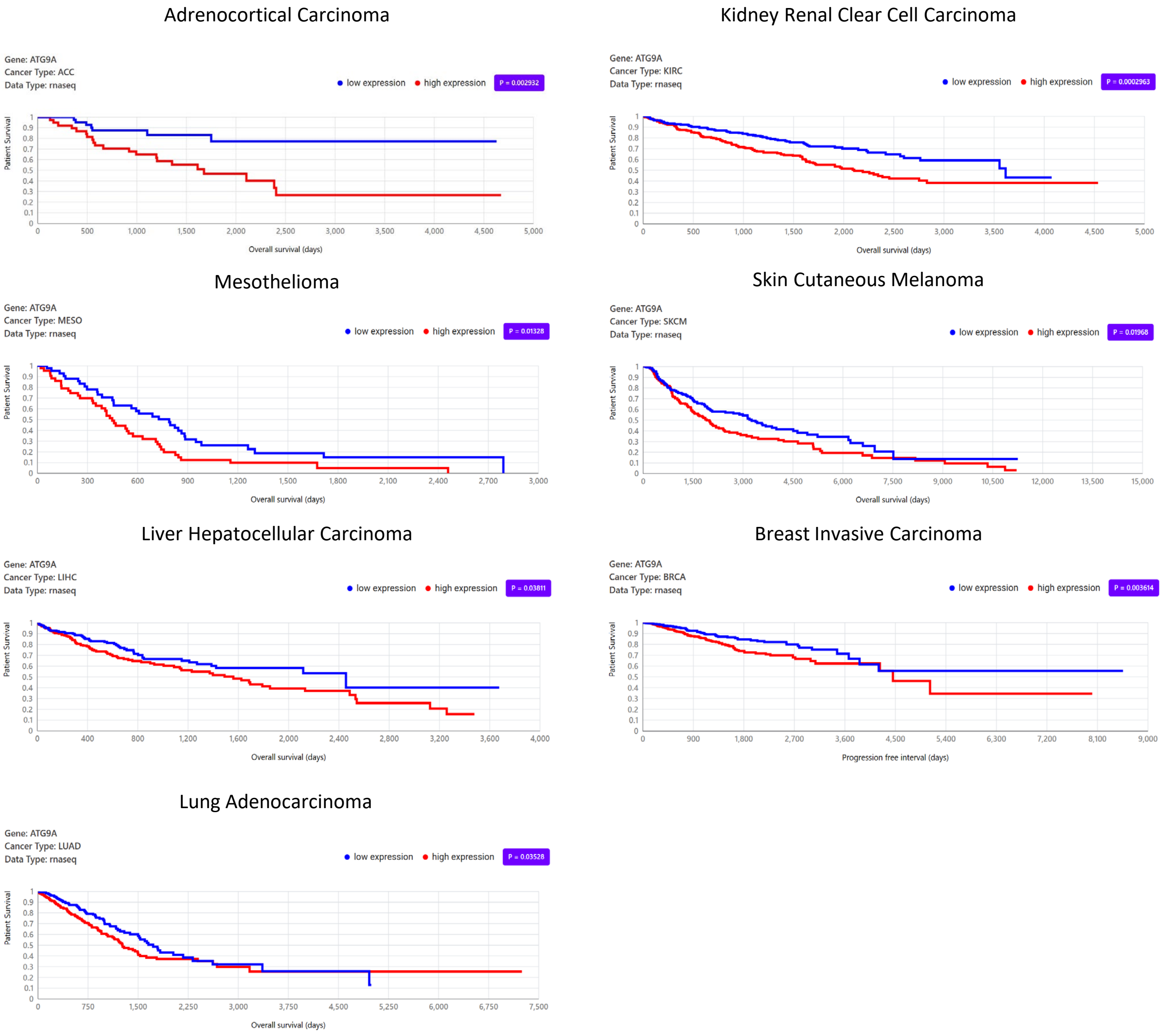

Supplementary Figure 4. Lower ATG9A mRNA (≥ median) is associated with better survival in several additional cancer types (related to Figure 2).

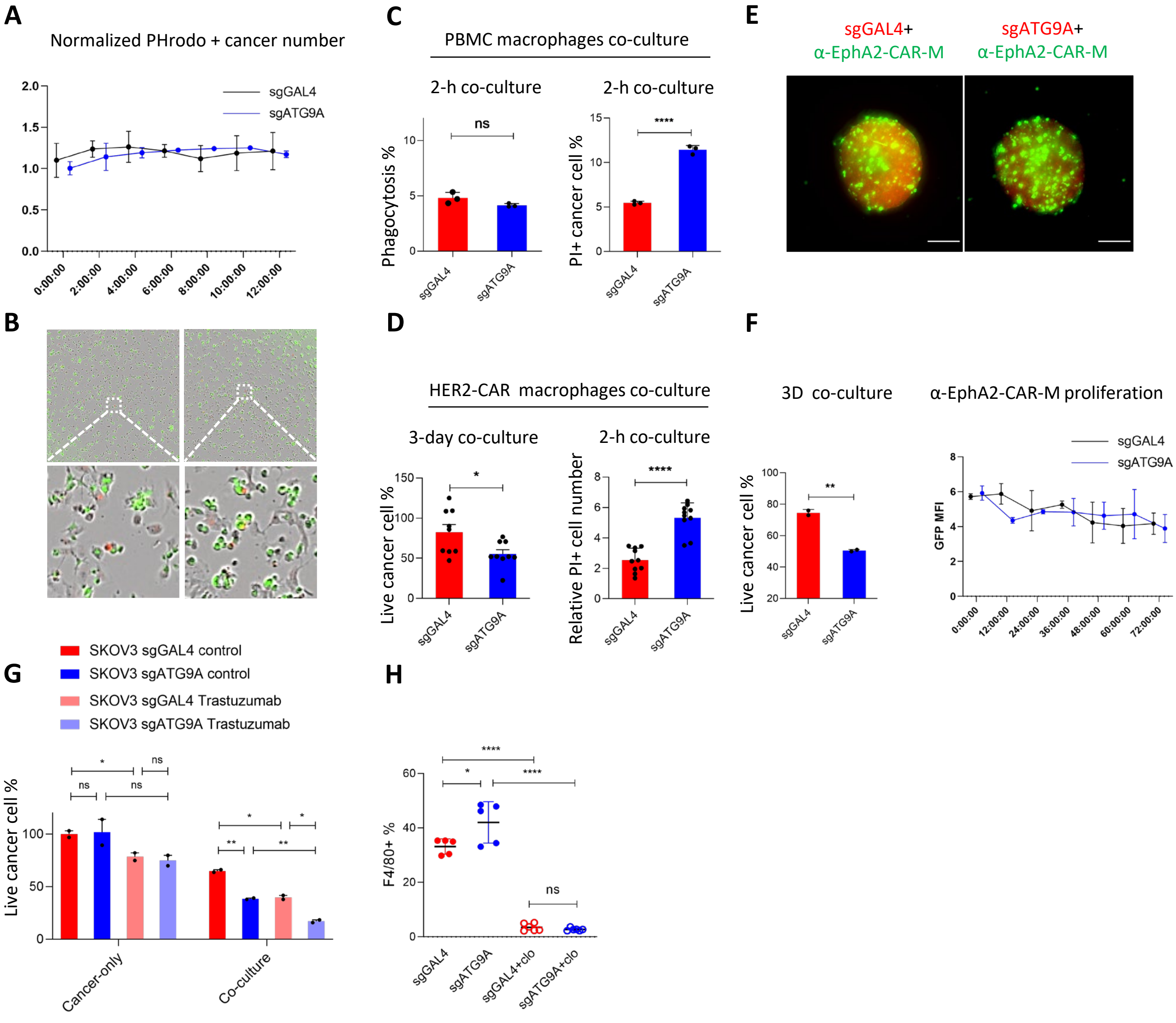

Supplementary Figure 5. ATG9A protects ovarian cancer cells from CAR-M-induced plasma membrane damage. (related to Figure 2 and 3).

(A) PHrodo-labeled sgGAL4 and sgATG9A OVCAR-8 cells were co-cultured with α-EphA2-CAR-Ms. PHrodo+ cell number were normalized by PHrodo+ cell number at 0 h (n = 2 biological replicates; mean ± SEM). (B) Representative images showing PHrodo+ OVCAR-8 cells engulfed by GFP+ CAR-Ms. (C) sgGAL4 and sgATG9A OVCAR-8 cells were co-cultured with α-EphA2-CAR-Ms for two hours. Phagocytosis % and PI+ cancer cell % were quantified by flow cytometry (n = 3 biological replicates; mean ± SEM). Statistical significance was determined using two-tailed unpaired Student's t-tests. (D) sgGAL4 and sgATG9A OVCAR-8 cells were co-cultured with PBMC-derived HER2-CAR-macrophages. Live cancer cell % was quantified by normalizing day-three cancer cell number in co-culture group by cancer-only group (n = 9 biological replicates, mean ± SEM). Relative PI+ cancer cell number was normalized by PI+ number at 0 h (n = 10, mean ± SEM). Statistical significance was determined using two-tailed unpaired Student's t-tests. (E-F) 3D co-culture of NL Red-labeled sgGAL4 or sgATG9A OVCAR-8 cells and GFP-labeled α-EphA2-CAR macrophages. CAR-M proliferation was quantified by GFP MFI over three days (n = 3 biological replicates; mean ± SEM). Statistical significance was determined using two-tailed unpaired Student's t-tests. Scale bar = 100 μm. (G) In vitro ADCC assay using HER2+ SKOV3 cell line (n = 2 biological replicates; mean ± SEM). Statistical significance was determined using two-way ANOVA with Tukey's multiple comparisons test. (H) Flow cytometry on F4/80 was performed to confirm macrophage depletion in clodronate-treated tumors (n= 5 tumors, mean ± SEM). Statistical significance was determined using two-way ANOVA with Tukey's multiple comparisons test. *P < 0.05; **P < 0.01; ***P < 0.001.

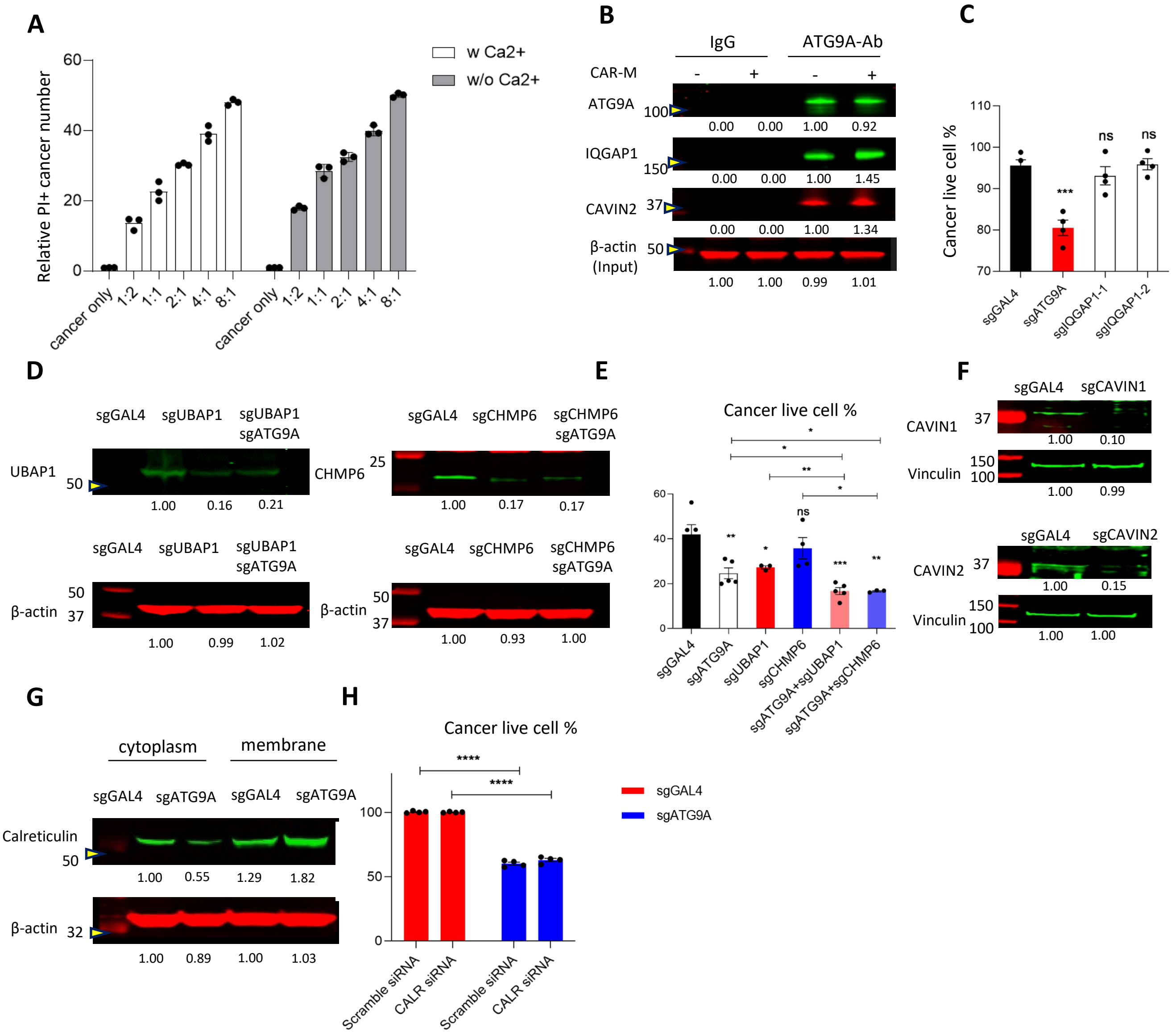

Supplementary Figure 6. Roles of ATG9A in other pathways involved in cancer cell response to macrophages (related to Figure 4 and 5).

(A) α-EphA2-CAR-macrophages were co-cultured with OVCAR-8 cells at different E:T ratios. Relative PI+ cell number was normalized by PI+ cell number at 0 h (n = 3 biological replicates; mean ± SEM). (B) Co-IP was performed to investigate proteins interacting with ATG9A. β-actin antibody was used as a loading control for the input samples. (C) Live cancer cell percentage was quantified by day-three cell count in co-cultured groups normalized by cell count in cancer-only groups (n = 4 biological replicates; mean ± SEM). Statistical significance was determined using two-tailed unpaired Student's t-tests. (D) Western blot results confirming the KO efficiency of UBAP1 and CHMP6. (E) Live cell percentage was calculated by cell count in co-culture with α-EphA2-CAR macrophages, normalized by cancer-only control (n = 3 biological replicates; mean ± SEM). Statistical significance was determined using one-way ANOVA with Dunnett's multiple comparisons test. (F) Western blot confirming the KO efficiency of CAVIN1 and CAVIN2. (G) Cytoplasm and membrane protein of control or KO OVCAR-8 cells were isolated to assess calreticulin expression. (H) IncuCyte co-culture experiment was performed using control or sgATG9A OVCAR-8 cells transfected with scramble siRNA or CALR siRNA. (n = 4 biological replicates; mean ± SEM). Statistical significance was determined using two-way ANOVA with Tukey's multiple comparisons test. *P < 0.05; **P < 0.01; ***P < 0.001.

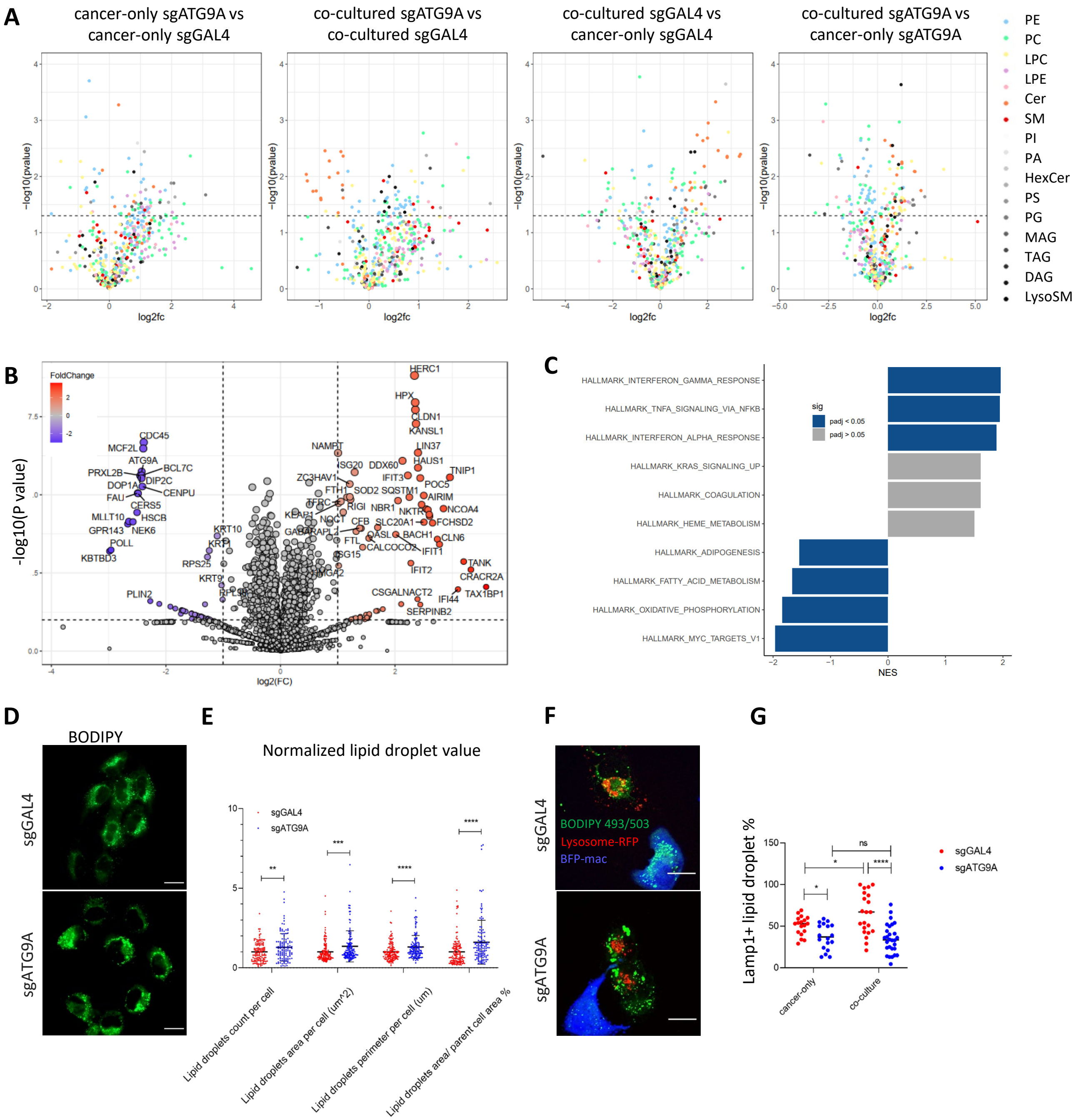

Supplementary Figure 7. ATG9A KO increases the number and size of lipid droplets in OVCAR-8 cells (related to Figure 6). (A) Differential abundance of lipid species between isolated membrane samples from control or KO OVCAR-8 cells from cancer-only samples or sorted from co-culture. Representative lipid species are depicted in different colors. (B) Mass spectrometry using control or sgATG9A OVCAR-8 cells sorted after three-day co-culture with α-EphA2-CAR macrophages. (C) Gene Set Enrichment Analysis (GSEA) using the Hallmark gene set. (D) BODIPY staining was performed using control and KO OVCAR-8 cells (n = 136 cells). Scale bar = 20 μm. (E) Lipid droplets count, area, perimeter, and lipid droplet area/parent cell area percentage were quantified by Nanolive (each dot represents a cell; mean ± SEM). Statistical significance was determined using two-tailed unpaired Student's t-tests for each LD feature. (F-G) BODIPY staining was performed using control and KO OVCAR-8 cells infected with Cellight Lysosome-RFP. Lysosome-RFP+ lipid droplet percentage was quantified. Each dot represents a single cell (n = 20 images). Scale bar = 20 μm. Statistical significance was determined using two-way ANOVA with Tukey's multiple comparisons test. *P < 0.05; **P < 0.01; ***P < 0.001.

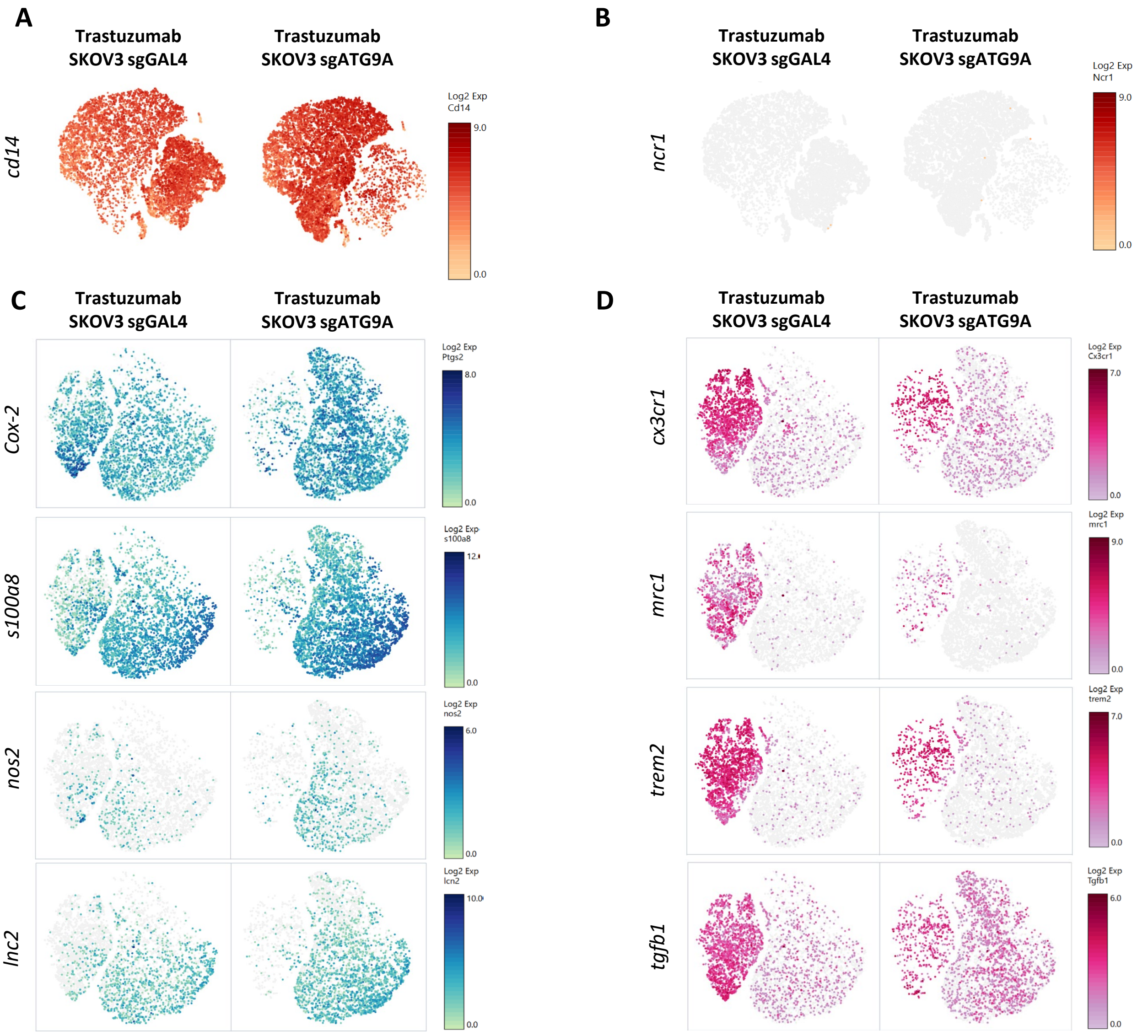

Supplementary Figure 8. Single-cell RNA-Seq reveals upregulated pro-inflammatory and reduced anti-inflammatory macrophage signatures in ATG9A KO tumors (related to Figure 9).

(A-B) t-SNE plots showing most cells are positive for the monocyte/macrophage marker *cd14* and negative for the NK cell marker *ncr1*. (C-D) tSNE plot shows clustering of cells after filtered out *cd14*- cells. Cell color specifies the expression distribution of pro-inflammatory (blue) or anti-inflammatory (red) macrophage markers based on gene expression.

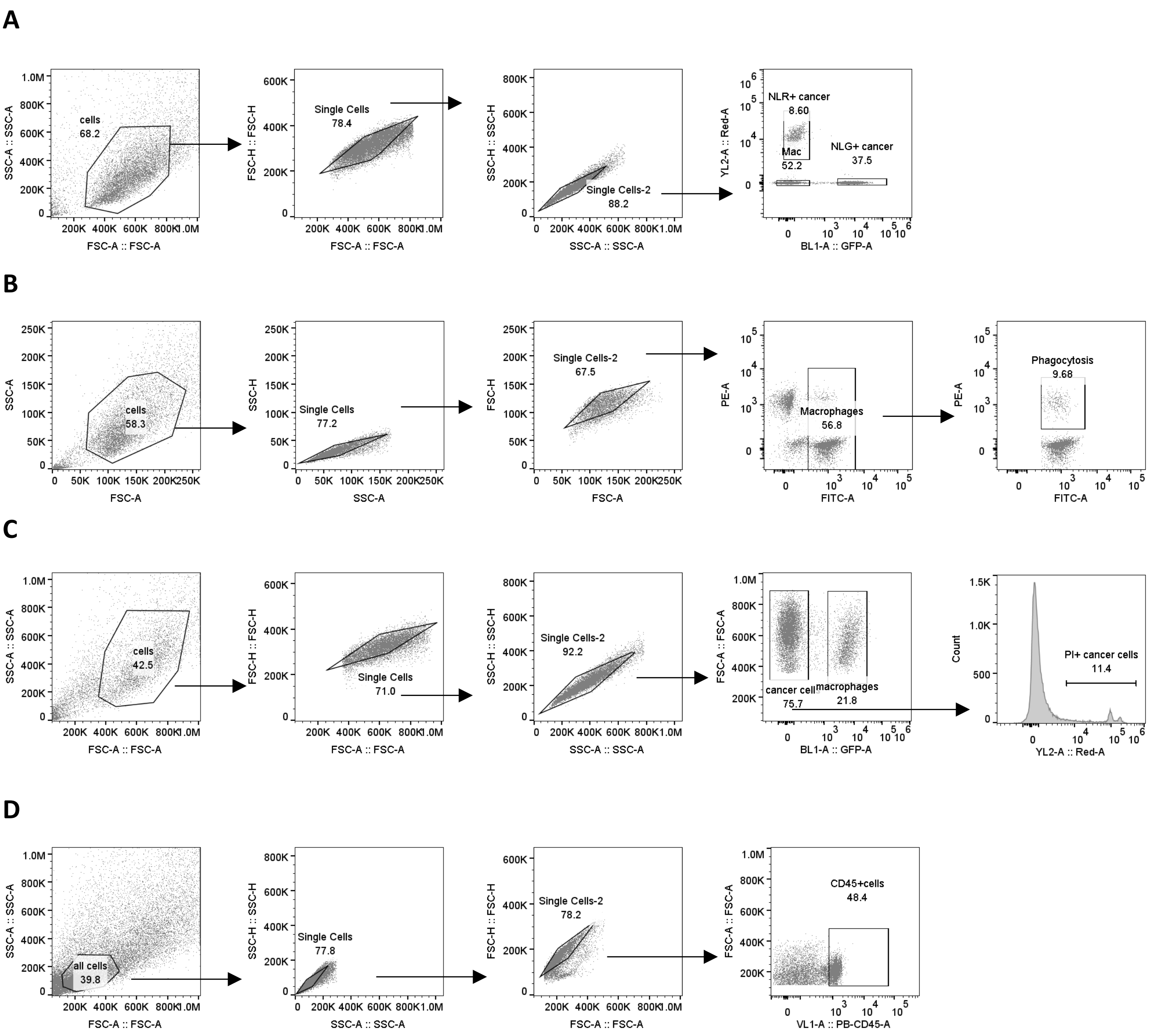

Supplementary Figure 9. Gating strategies used for Flow cytometry or cell sorting. (related to Figure 2 and Figure 9).
(A) Gating strategy for competition assay presented on Figure 2. (B) Gating strategy for phagocytosis assay presented on Figure 2. (C) Gating strategy for PI plasma membrane integrity assay presented on Figure 2. (D) Gating strategy for sorting CD45+ mouse immune cells for single-cell RNA-Seq experiment presented on Figure 9.

<table>
<tr><th>Models</th><th colspan="2">α-EphA2-CAR-THP-1-macrophages</th><th>α-CD19-CAR-THP-1-macrophages</th><th>PBMC macrophages</th><th>PBMC-derived HER2-CAR-macrophages</th></tr>
<tr><td rowspan="2">co-culture CRISPR screen</td><td colspan="2">OVCAR-8</td><td>OVCAR-8</td><td rowspan="2"></td><td rowspan="2"></td></tr>
<tr><td colspan="2">Figure 1C</td><td>Figure 1D</td></tr>
<tr><td rowspan="2">Screen validation</td><td>OVCAR-8</td><td>SKOV3</td><td>OVCAR-8</td><td>OVCAR-8</td><td rowspan="2"></td></tr>
<tr><td>Figure 2A</td><td>Figure S2C</td><td>Figure 2A</td><td>Figure S2E</td></tr>
<tr><td rowspan="2">In vitro killing assay</td><td>OVCAR-8</td><td>SKOV3</td><td>OVCAR-8</td><td>OVCAR-8</td><td>SKOV3</td></tr>
<tr><td>Figure 2B-C</td><td>Figure S2C</td><td>Figure S2F</td><td>Figure S5C</td><td>Figure S5D</td></tr>
<tr><td rowspan="2">In vitro PM integrity assay</td><td colspan="2">OVCAR-8</td><td rowspan="2"></td><td>OVCAR-8</td><td>SKOV3</td></tr>
<tr><td colspan="2">Figure 2F</td><td>Figure S5C</td><td>Figure S5D</td></tr>
<tr><td>In vivo subcutaneous SKOV3 tumor model</td><td colspan="5">SKOV3 + endogenouse mouse macrophages</td></tr>
<tr><td>In vivo intraperineal SKOV3 tumor model</td><td colspan="5">SKOV3 + α-EphA2-CAR-THP-1-macrophages</td></tr>
</table>

Supplementary Table 1. Summary of cell lines and animal models used in this study.
