# Supplementary material for: Exploitable mechanisms of antibody and CAR mediated macrophage cytotoxicity
Source: Nat Commun. 2025 Jul 1;16:5616. doi: 10.1038/s41467-025-60745-x (PMC12216399; doi:10.1038/s41467-025-60745-x)
Supplement: Supplementary file 2 — Description of Additional Supplementary Files [file 41467_2025_60745_MOESM2_ESM.pdf]

Description of Additional Supplementary Files

File name: Supplementary Data 1
Description: Table listing the CRISPR library composition, including sgRNA sequences used in the pooled screen.

File name: Supplementary Data 2
Description: Results from the CRISPR screen performed in OVCAR-8 cells with EphA2-CAR-Macrophages.

File name: Supplementary Data 3
Description: Results from the CRISPR screen in co-cultures of $CD19^{+}$ cells and OVCAR-8 cells with CD19-CAR-Macrophages.

File name: Supplementary Data 4
Description: Lipidomics datasets comparing single and co-culture conditions of control and KO cells.

File name: Supplementary Data 5
Description: List of key reagents and antibodies used in the study, including vendors, catalog numbers, and working dilutions.
