# Supplementary material for: Exploitable mechanisms of antibody and CAR mediated macrophage cytotoxicity
Source: Nat Commun. 2025 Jul 1;16:5616. doi: 10.1038/s41467-025-60745-x (PMC12216399; doi:10.1038/s41467-025-60745-x)
Supplement: Supplementary file 8 — Reporting Summary [file 41467_2025_60745_MOESM8_ESM.pdf]

nature portfolio

Corresponding author(s): Carl J. DeSelm
Felix Y. Feng

Last updated by author(s): 05/13/2025

Reporting Summary

Nature Portfolio wishes to improve the reproducibility of the work that we publish. This form provides structure for consistency and transparency in reporting. For further information on Nature Portfolio policies, see our Editorial Policies and the Editorial Policy Checklist.

Statistics

For all statistical analyses, confirm that the following items are present in the figure legend, table legend, main text, or Methods section.

n/a	Confirmed	
☐	☒	The exact sample size (*n*) for each experimental group/condition, given as a discrete number and unit of measurement
☐	☒	A statement on whether measurements were taken from distinct samples or whether the same sample was measured repeatedly
☐	☒	The statistical test(s) used AND whether they are one- or two-sided<br>*Only common tests should be described solely by name; describe more complex techniques in the Methods section.*
☐	☒	A description of all covariates tested
☐	☒	A description of any assumptions or corrections, such as tests of normality and adjustment for multiple comparisons
☐	☒	A full description of the statistical parameters including central tendency (e.g. means) or other basic estimates (e.g. regression coefficient) AND variation (e.g. standard deviation) or associated estimates of uncertainty (e.g. confidence intervals)
☐	☒	For null hypothesis testing, the test statistic (e.g. *F*, *t*, *r*) with confidence intervals, effect sizes, degrees of freedom and *P* value noted<br>*Give P values as exact values whenever suitable.*
☒	☐	For Bayesian analysis, information on the choice of priors and Markov chain Monte Carlo settings
☒	☐	For hierarchical and complex designs, identification of the appropriate level for tests and full reporting of outcomes
☐	☒	Estimates of effect sizes (e.g. Cohen's *d*, Pearson's *r*), indicating how they were calculated

Our web collection on statistics for biologists contains articles on many of the points above.

Software and code

Policy information about availability of computer code

Data collection	ZEN 3.9 software (ZEISS), llumina NextSeq platform, Incucyte S3 Software (Sartorius), Attune NXT Software Version 3.1 (Thermo Fisher), Orbitrap Tribrid ID-X mass spectrometer (Thermo Scientific)
Data analysis	GraphPad Prism 6.05 (GraphPad Software, Inc.), FlowJo v.10 (FlowJo LLC), Fiji software (ImageJ), Excel (Microsoft), Cell Ranger software v8.0.0., Loupe Browser v8.0.0., Incucyte S3 Software (Sartorius)

For manuscripts utilizing custom algorithms or software that are central to the research but not yet described in published literature, software must be made available to editors and reviewers. We strongly encourage code deposition in a community repository (e.g. GitHub). See the Nature Portfolio guidelines for submitting code & software for further information.

Data

Policy information about availability of data

All manuscripts must include a data availability statement. This statement should provide the following information, where applicable:

- Accession codes, unique identifiers, or web links for publicly available datasets
- A description of any restrictions on data availability
- For clinical datasets or third party data, please ensure that the statement adheres to our policy

The processed sequencing data in this paper have been deposited into the NCBI GEO database: GSE266329 (token: mlmjoksabvqfpej). Proteomics data have been deposited into PRIDE: PXD053872 (token: qBQzN7eJ9E9b)

Research involving human participants, their data, or biological material

Policy information about studies with human participants or human data. See also policy information about sex, gender (identity/presentation), and sexual orientation and race, ethnicity and racism.

Reporting on sex and gender	N/A
Reporting on race, ethnicity, or other socially relevant groupings	N/A
Population characteristics	N/A
Recruitment	N/A
Ethics oversight	N/A

Note that full information on the approval of the study protocol must also be provided in the manuscript.

Field-specific reporting

Please select the one below that is the best fit for your research. If you are not sure, read the appropriate sections before making your selection.

- [x] Life sciences
- [ ] Behavioural & social sciences
- [ ] Ecological, evolutionary & environmental sciences

For a reference copy of the document with all sections, see nature.com/documents/nr-reporting-summary-flat.pdf

Life sciences study design

All studies must disclose on these points even when the disclosure is negative.

Sample size	Sample size was calculated by estimating the confidence interval and margin of error, or experiments were ran in at least duplicate/triplicate.
Data exclusions	No relevant data were excluded. A priori criteria for exclusion were developed.
Replication	All experiments were conducted at least in biological duplicate or triplicate. When available and possible we used macrophages derived from multiple normal donors and different cell lines. All attempts at replication were successful.
Randomization	Randomization was not relevant to this work. For in vivo models, baseline tumor burden was verified as equivalent (i.e. there was no statistically significant difference amongst the groups via ANOVA) between treatment groups.
Blinding	Blinding analysis was implented in quantification of tumor cells in immunohistochemistry sections.

Reporting for specific materials, systems and methods

We require information from authors about some types of materials, experimental systems and methods used in many studies. Here, indicate whether each material, system or method listed is relevant to your study. If you are not sure if a list item applies to your research, read the appropriate section before selecting a response.

Materials & experimental systems

n/a	Involved in the study	
[ ]	[x]	Antibodies
[ ]	[x]	Eukaryotic cell lines
[x]	[ ]	Palaeontology and archaeology
[ ]	[x]	Animals and other organisms
[x]	[ ]	Clinical data
[x]	[ ]	Dual use research of concern
[x]	[ ]	Plants

Methods

n/a	Involved in the study	
[x]	[ ]	ChIP-seq
[ ]	[x]	Flow cytometry
[x]	[ ]	MRI-based neuroimaging

Antibodies

Antibodies used	β-Actin (8H10D10) Mouse mAb CST 3700<br>Vinculin (E1E9V) XP® Rabbit mAb CST 13901s<br>Atg9A (D4O9D) Rabbit mAb CST 13509S<br>UBAP1 Polyclonal antibody proteintech 12385-1-AP

Anti-SEL1L antibody abcam ab78298
SCAP Antibody CST 13102
RFP Monoclonal Antibody (RF5R) invitrogen MA5-15257
CHMP6 Polyclonal Antibody proteintech 16278-1-AP
IQGAP1 Monoclonal Antibody (AF1) invitrogen 33-8900
Beclin-1 (D40C5) Rabbit mAb CST 3495
LC3A/B (D3U4C) XP® Rabbit mAb CST 12741
Atg5 (D5F5U) Rabbit mAb CST 12994
Atg12 (D88H11) Rabbit mAb CST 4180
Atg16L1 (D6D5) Rabbit mAb CST 8089
Atg7 (D12B11) Rabbit mAb CST 8558
Atg3 Antibody CST 3415
Atg2A Antibody CST 15011
SQSTM1/p62 Antibody CST 5114
Caveolin-2 (D4A6) XP® Rabbit mAb CST 8522
Caveolin-1 (D46G3) XP® Rabbit mAb CST 3267
CAVIN1 antibody aviva ARP72430_P050
CAVIN2 antibody aviva ARP76755_P050
Claudin-1 (D5H1D) XP® Rabbit mAb CST 13255T
Calreticulin Antibody CST 2891
Syndecan 2 Polyclonal Antibody invitrogen 36-6200
Syndecan 4 Antibody CST 12236
Recombinant Anti-ATG9A antibody [EPR2450(2)] Abcam ab108338
Fatty Acid Synthase (C20G5) Rabbit mAb CST 3180
Lipin 1 (D2W9G) Rabbit mAb CST 14906
ACSL1 (D2H5) Rabbit mAb CST 9189
Perillipin-2 rabbit mAb CST 95109S
Anti-CX3CR1 antibody [8E10.D9] Abcam ab184678
Anti-iNOS antibody Abcam ab15323
Anti-SDPR CAVIN2 Antibody Boster A31733
ATG9A Monoclonal Antibody (14F2 8B1), Alexa Fluor™ 647 Fisher Scientific MA1-149-A647
Caveolin 1 Monoclonal Antibody (7C8) Invitrogen MA3-600
Caveolin-2 (D4A6) XP® Rabbit mAb #8522 CST 8522
CD107a (LAMP-1) Monoclonal Antibody (eBio1D4B (1D4B)), eBioscience™ Invitrogen 14-1071-82
CD11b Monoclonal Antibody (M1/70), Alexa Fluor™ 488, eBioscience™ Fisher Scientific 15371980
CD206/MRC1 (E6T5J) XP® Rabbit mAb 24595 CST 24595
Ceramide monoclonal antibody Enzo MID 15B4
DyLight 594 Phalloidin CST 12877s
F4/80 (D2S9R) XP® Rabbit mAb 70076 CST 70076
F4/80 Monoclonal Antibody (BM8), eFluor™ 570 Invitorgen 41-4801-82
Goat anti-Mouse IgG (H+L) Cross-Adsorbed Secondary Antibody, Alexa Fluor 594 ThermoFisher A11005
Goat anti-Mouse IgG, IgM (H+L) Secondary Antibody, Alexa Fluor™ 488 ThermoFisher A10680
Goat anti-Rabbit IgG (H+L) Cross-Adsorbed Secondary Antibody, Alexa Fluor™ 488 ThermoFisher A11008
Goat anti-Rabbit IgG (H+L) Cross-Adsorbed Secondary Antibody, Alexa Fluor™ 568 ThermoFisher A11011
Goat anti-Rabbit IgG (H+L) Cross-Adsorbed Secondary Antibody, Alexa Fluor™ 594 ThermoFisher A11012
IQGAP1 Monoclonal Antibody (AF1) Invitorgen 33-8900
LC3A/B (D3U4C) XP® Rabbit mAb 12741 CST 12741
Paxillin Monoclonal Antibody (5H11) Invitrogen AHO0492
Recombinant Alexa Fluor® 647 Anti-ATG9A antibody Abcam ab206253
Recombinant Anti-CSF-1-R antibody Abcam ab254357
Recombinant Anti-CX3CR1 antibody [EPR24267-2] Abcam ab308613
Recombinant Anti-F4/80 antibody [EPR26545-166] Abcam ab300421
Recombinant Anti-iNOS antibody [SP126] Abcam ab115819
Pacific Blue™ anti-human CD11b Antibody biolegend 301315
Pacific Blue™ Mouse IgG1, κ Isotype Ctrl biolegend 981812
Pacific Blue™ anti-human CD14 Antibody biolegend 325616
Human CD24 Antibody R&D Systems AF5247
Human CD47 Antibody R&D Systems AF4670
PE-human-CD206 biolegend 321105
PE-human-CD163 biolegend 326505
Pacific Blue anti-human CD47 biolegend 323128
FITC anti-human CD24 biolegend 311104
FITC anti-human β2-microglobulin Antibody biolegend 395705
PE anti-human CD274/PD-L1 biolegend 393607
HLA-ABC Monoclonal Antibody (W6/32), APC invitrogen 17-9983-42
APC anti-human β2-microglobulin Antibody biolegend 316311
FITC anti-human β2-microglobulin Antibody biolegend 395706
Pacific Blue™ anti-human HLA-A,B,C Antibody biolegend 311418

	APC anti-human CD206 (MMR) Antibody biolegend 321109<br>APC anti-human CD80 Antibody biolegend 305219<br>Human EphA2 alexa fluor-488 R&D Systems FAB3035G<br>APC anti-human CD19 Antibody biolegend 363006<br>CD11b Monoclonal Antibody (M1/70), Alexa Fluor™ 488 ThermoScientific 53-0112-82<br>FITC anti-mouse F4/80 Recombinant Biolegend 157310<br>Biolegend Pacific Blue™ anti-mouse CD45 Biolegend 157212<br>APC anti-Nos2 (iNOS) Antibody<br>Biolegend 696808<br>Brilliant Violet 510™ anti-mouse CD183 (CXCR3) Antibody<br>Biolegend 155919<br>Alexa Fluor® 647 AffiniPure™ F(ab')$_2$ Fragment Goat Anti-Mouse IgG, F(ab')$_2$ fragment specific Jackson ImmunoResearch 115-605-006<br>PE-Labeled Monoclonal Anti-FMC63 Antibody, Mouse IgG1 (Y45) (Site-specific conjugation) (Preservative free)DMF Acro Biosystems FM3-HPY53<br>PE anti-human CX3CR1 Antibody Biolegend 355703<br>Human IgG Isotype Control [Unconjugated] NOVUS 1-001-A<br>Human IFN-gamma ab R&D Systems MAB2851-SP<br>Human TNF-a ab R&D Systems MAB210-SP<br>InVivoSIM anti-human HER2 (Trastuzumab Biosimilar) BioXcell SIM0005<br>InVivoPlus human IgG1 isotype control BioXcell BP0297
Validation	Antibodies were validated using positive and negative cells or isotype controls. Validation reports were provided by the antibody manufacturers (BioLegend). Appropriate compensation controls were used for every experiment. The BD Fusion and Attune Nxt were calibrated daily using CS&T beads (BD Biosciences) or manufacturer recommended methods.

Eukaryotic cell lines

Policy information about cell lines and Sex and Gender in Research

Cell line source(s)	Cell lines were purchased from and validated by the American Type Culture Collection (ATCC).
Authentication	Cell lines were authenticated as by routine practice by UC Berkeley DNA Sequencing Facility.
Mycoplasma contamination	Cell lines were tested for the presence of mycoplasma contamination (MycoAlert™ Mycoplasma Detection Kit, LT07-318, Lonza, Basel, Switzerland). All the cell line were negative for mycoplsma.
Commonly misidentified lines<br>(See ICLAC register)	None of the cell lines used in this manuscript are listed in the ICLAC Database of Cross-contaminated or Misidentified Cell Lines (Version 8.0).

Animals and other research organisms

Policy information about studies involving animals; ARRIVE guidelines recommended for reporting animal research, and Sex and Gender in Research

Laboratory animals	NOD.Cg-Prkdc scid Il2rg tm1Wjl /SzJ (NSG) mice were purchased from Jackson Laboratories.
Wild animals	The study did not involve wild animals.
Reporting on sex	Female mice were used, age 6-8 weeks.
Field-collected samples	The study did not involve samples collected from the field.
Ethics oversight	All in vivo studies were conducted under IACUC approved protocols under established policies at UCSF.

Note that full information on the approval of the study protocol must also be provided in the manuscript.

Plants

Seed stocks	N/A
Novel plant genotypes	N/A
Authentication	N/A

Flow Cytometry

Plots

Confirm that:

- [x] The axis labels state the marker and fluorochrome used (e.g. CD4-FITC).
- [x] The axis scales are clearly visible. Include numbers along axes only for bottom left plot of group (a 'group' is an analysis of identical markers).
- [x] All plots are contour plots with outliers or pseudocolor plots.
- [x] A numerical value for number of cells or percentage (with statistics) is provided.

Methodology

Sample preparation	Standard flow cytometry protocol was used in this study. Cells were washed before and after staining, and Fc block was used when staining Fc receptor expressing cells (macrophages, PBMC's, THP-1s). Samples were acquired on an Fusion (BD) or an Attune Nxt. Compensation was performed with every experiment and the instrument was calibrated daily using CS&T beads. Sample preparation details on individual experiments are in the Methods section.
Instrument	Flow cytometry was performed on Fusion (Becton-Dickinson) or Attune NxT (Thermo Fisher).
Software	FCS files were analyzed with FlowJo X 10.7.2.
Cell population abundance	Target cell lines were sorted for > 99% NucLight Red positivity on a BD Fusion Cell Sorter (BD Biosciences).THP-1's were sorted for >905% CAR positivity on a BD Fusion cell sorter. Purity was determined by flow cytometry during and after the sort. Purity was periodically checked if cells were kept in culture.
Gating strategy	Generally, FACS gating was performed as follows: FSC/SSC -> Singlets -> Live cells (L/D NearIR negative) -> gating of interest. The appropriate negative control was used for generating gates of interest. For co-culture assays, gating was performed as follows: FSC/SSC -> Singlets> GFP+ -> GFP+/NucLight Red+. Gates were drawn using macrophages alone as a control.

- [x] Tick this box to confirm that a figure exemplifying the gating strategy is provided in the Supplementary Information.
